# PWSC: a novel clustering method based on polynomial weight-adjusted sparse clustering for sparse biomedical data and its application in cancer subtyping

**DOI:** 10.1186/s12859-023-05595-4

**Published:** 2023-12-21

**Authors:** Xiaomeng Zhang, Hongtao Zhang, Zhihao Wang, Xiaofei Ma, Jiancheng Luo, Yingying Zhu

**Affiliations:** 1 Department of Nephrology, Tongji Hospital, Tongji Medical College, Huazhong University of Science and Technology, Wuhan, 430030 Hubei Province China; 2 School of Mathematics and Statistics, Wuhan University, Wuhan, 430070 Hubei Province China; 3 Department of Oncology, Tongji Hospital, Tongji Medical College, Huazhong University of Science and Technology, Wuhan, 430030 Hubei Province China

**Keywords:** Hierarchical clustering, Polynomial weight, Consensus clustering, Sparse biomedical data

## Abstract

**Background:**

Clustering analysis is widely used to interpret biomedical data and uncover new knowledge and patterns. However, conventional clustering methods are not effective when dealing with sparse biomedical data. To overcome this limitation, we propose a hierarchical clustering method called polynomial weight-adjusted sparse clustering (PWSC).

**Results:**

The PWSC algorithm adjusts feature weights using a polynomial function, redefines the distances between samples, and performs hierarchical clustering analysis based on these adjusted distances. Additionally, we incorporate a consensus clustering approach to determine the optimal number of classifications. This consensus approach utilizes relative change in the cumulative distribution function to identify the best number of clusters, resulting in more stable clustering results. Leveraging the PWSC algorithm, we successfully classified a cohort of gastric cancer patients, enabling categorization of patients carrying different types of altered genes. Further evaluation using Entropy showed a significant improvement (*p* = 2.905e−05), while using the Calinski–Harabasz index demonstrates a remarkable 100% improvement in the quality of the best classification compared to conventional algorithms. Similarly, significantly increased entropy (*p* = 0.0336) and comparable CHI, were observed when classifying another colorectal cancer cohort with microbial abundance. The above attempts in cancer subtyping demonstrate that PWSC is highly applicable to different types of biomedical data. To facilitate its application, we have developed a user-friendly tool that implements the PWSC algorithm, which canbe accessed at http://pwsc.aiyimed.com/.

**Conclusions:**

PWSC addresses the limitations of conventional approaches when clustering sparse biomedical data. By adjusting feature weights and employing consensus clustering, we achieve improved clustering results compared to conventional methods. The PWSC algorithm provides a valuable tool for researchers in the field, enabling more accurate and stable clustering analysis. Its application can enhance our understanding of complex biological systems and contribute to advancements in various biomedical disciplines.

## Introduction

In biomedical data processing, cluster analysis is an essential tool that can be utilized to classify and predict various types of biological molecule data [1].By grouping similar data points together, cluster analysis forms clusters with distinct features that can differentiate different biological entities, including gene sequences, proteins, and more [2–5]. Through the use of cluster analysis, vast amounts of biological data can be organized and analyzed in a meaningful way, leading to more precise biomedical information [6]. Additionally, cluster analysis can also be employed to analyze the structure and function of biological systems, providing new approaches and perspectives for biomedical research [7].

Commonly used methods in cluster analysis include K-means clustering and hierarchical clustering [8, 9]. The former involves dividing data points into K groups, with the center of each group being the average value of all data points within it [10, 11]. The latter method involves gradually grouping data points based on their similarities, forming a tree-like structure [12, 13]. Additionally, there have been some clustering methods specifically designed for certain problems. For example, Kath Nicholls et al. proposed the Biclustering algorithm to address the issue that genes cluster differently in heterogeneous samples and cannot achieve effective clustering [14]. Juan Wang et al. improved the clustering quality of multi-cancer samples based on gene expression data by applying the graph regularized low-rank representation under symmetric and sparse constraints (sgLRR) method [15].

However, the data in the biomedical domain is often high-dimensional and sparse, primarily due to the complexity of multiple biomolecules, tissues, and organs in living organisms [16]. The high dimensionality and sparsity of biomedical data result in samples being sparsely distributed in a high-dimensional clustering space, making it challenging for conventional clustering methods to effectively capture similarities and affinities between samples [17, 18]. This can lead to issues such as overfitting or underfitting [19].

To address the problem that conventional clustering methods are difficult to handle due to the sparsity of biomedical data, we propose a new clustering algorithm. This algorithm recalculates the distances between samples by adjusting the weights of features, and performs clustering analysis based on this. At the same time, we use a consensus clustering method to select the optimal number of classifications, thus obtaining the most stable clustering results. With this integrated approach, we have successfully achieved effective clustering of biomedical data with good classification results, avoiding the overfitting or oversimplification problems that can occur in conventional methods.

## Methods

### Polynomial weight-adjusted sparse clustering

We redefined the distances between samples based on the hierarchical clustering method [12, 13].

By reading the data, we can obtain the following sparse matrix, the rows of which represent the performance values of one of its features in the sample and the columns represent the performance values of different features of one of the samples.

1 $$D = \left[ {\begin{array}{*{20}c} {\begin{array}{*{20}c} {d_{11} } & {d_{12} } \\ {d_{21} } & {d_{22} } \\ \end{array} } & \cdots & {\begin{array}{*{20}c} {d_{1n} } \\ {d_{2n} } \\ \end{array} } \\ \vdots & \ddots & \vdots \\ {\begin{array}{*{20}c} {d_{m1} } & {d_{m2} } \\ \end{array} } & \cdots & {d_{mn} } \\ \end{array} } \right] \in R^{mn}$$

We process this sparse matrix and count the frequency of its features in each row in the sample to obtain $\{ \eta_{1} ,\eta_{2} \ldots \eta_{m} \}$, and next, we build the polynomial:

2 $$P_{n}^{i} \left( {\eta_{i} } \right) = a_{n} \eta_{i}^{n} + a_{n - 1} \eta_{i}^{n - 1} + \cdots + a_{1} \eta_{i}^{1} + a_{0} ,$$

With the help of that established polynomial, we re-establish the weights $\left\{ { \overline{\eta }_{1} ,\overline{\eta }_{2} \ldots \overline{\eta }_{m} } \right\}$:

3 $$\overline{\eta }_{i} = \frac{{P_{n}^{i} \left( {\eta_{i} } \right)}}{{\mathop \sum \nolimits_{k = 1}^{m} P_{n}^{k} \left( {\eta_{k} } \right)}},$$

We adjust the weights of the sparse matrix to obtain a correction matrix $\overline{D}$, expressed as follows:

4 $$\overline{D}\left( {i , \cdot } \right) = D\left( {i , \cdot } \right) \times \overline{\eta }_{i} ,$$

5 $$\overline{D} = \left[ {\begin{array}{*{20}c} {\begin{array}{*{20}c} {d_{11} \overline{\eta }_{1} } & {d_{12} \overline{\eta }_{1} } \\ {d_{21} \overline{\eta }_{2} } & {d_{22} \overline{\eta }_{2} } \\ \end{array} } & \cdots & {\begin{array}{*{20}c} {d_{1n} \overline{\eta }_{1} } \\ {d_{2n} \overline{\eta }_{2} } \\ \end{array} } \\ \vdots & \ddots & \vdots \\ {\begin{array}{*{20}c} {d_{m1} \overline{\eta }_{m} } & {d_{m2} \overline{\eta }_{m} } \\ \end{array} } & \cdots & {d_{mn} \overline{\eta }_{m} } \\ \end{array} } \right] ,$$

After that, we perform hierarchical clustering on the corrected sparse matrix using the method of sum of squares of differences, with the algorithm shown as follows:

**Algorithm 1:**
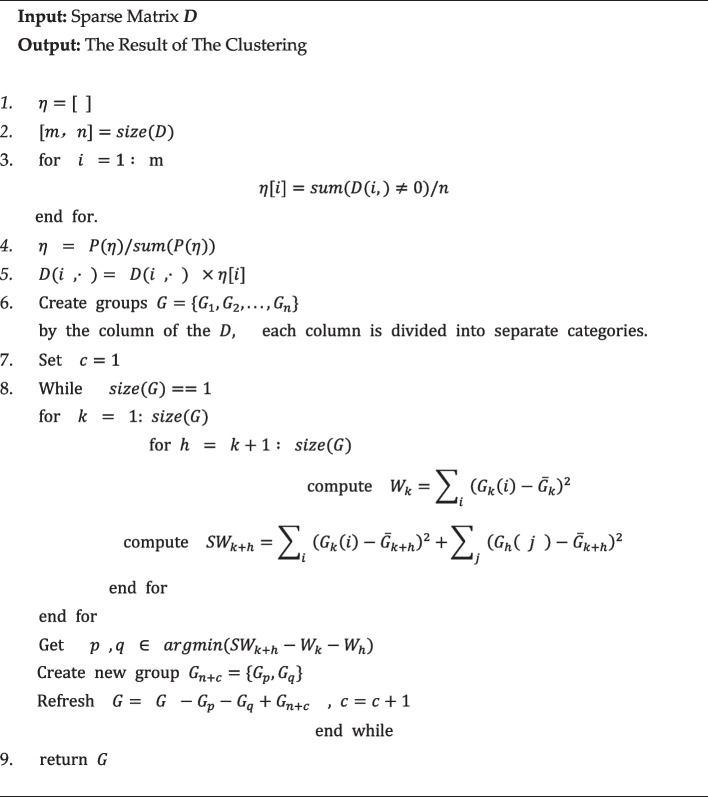
**PWSC (Polynomial Weight-adjusted Sparse Clustering)**

### Consensus clustering

The Monti consensus clustering algorithm is a well-known method for determining the number of clusters, *K*, in a dataset of *N* points [20, 21]. This algorithm involves resampling and clustering the data for each *K*, resulting in an *N* × *N* consensus matrix that indicates how often pairs of samples were clustered together. A perfectly stable matrix would contain only zeros and ones, indicating that all sample pairs either always clustered together or never did. By comparing the stability of the consensus matrices for different *K* values, the optimal *K* can be determined.

To be more precise, let $D = \left\{ {e^{1} , e^{2} , \ldots ,e^{N} } \right\}$ be the set of points to cluster, and let $D^{1} , D^{2} , \ldots , D^{H}$ be the H perturbed datasets resulting from resampling the original data. Let $M^{h}$ be the *N* × *N* connectivity matrix obtained by clustering $D^{h}$, with entries defined as follows:

6 $$M^{h} \left( {i,j} \right) = \left\{ {\begin{array}{*{20}l} {1,} \hfill & {\quad {\text{if points i and j belong to the same cluster}},} \hfill \\ {0,} \hfill & {\quad {\text{otherwise}}.} \hfill \\ \end{array} } \right.$$

Let $U^{h}$ be the *N* × *N* indicator matrix with $\left( {i,j} \right)$ entry equal to 1 if points i and j are in the same perturbed dataset $D^{h}$, and 0 otherwise. This matrix is used to keep track of which samples were selected during each resampling iteration for the normalization step. Thconsensus matrix *C* for a given *K* is defined as the normalized sum of all connectivity matrices of all the perturbed datasets:

7 $$C\left( {i,j} \right) = \frac{{\mathop \sum \nolimits_{h = 1}^{H} M^{h} \left( {i,j} \right)}}{{\mathop \sum \nolimits_{h = 1}^{H} U^{h} \left( {i,j} \right)}}$$

In other words, the entry $\left( {i,j} \right)$ in the consensus matrix is the number of times points i and j were clustered together, divided by the total number of times they were selected together. The consensus matrix is symmetric, and each element falls in the range [0,1]. A consensus matrix is calculated for each *K* value to be tested, and the stability of each matrix is assessed to determine the optimal *K*. One way to measure the stability of the Kth consensus matrix is by examining its cumulative distribution function (CDF) curve.

### Determination of optimal classification

Şenbabaoğlu et al. discovered that the original delta K metric used in the Monti algorithm performed poorly when deciding on the optimal number of clusters, and proposed a superior metric for measuring the stability of consensus matrices using their CDF curves [21]. In the CDF curve of a consensus matrix, the lower left portion represents sample pairs that are rarely clustered together, while the upper right portion represents those that are almost always clustered together. The middle segment represents those with ambiguous assignments in different clustering runs. The proportion of ambiguous clustering (PAC) score measures the fraction of sample pairs with consensus indices falling in the interval $\left( {u_{1} , u_{2} } \right) \in \left[ {0, 1} \right]$, where $u_{1}$ is a value close to 0 and $u_{2}$ is a value close to 1. A low value of PAC indicates a flat middle segment and a low rate of discordant assignments across permuted clustering runs. The optimal number of clusters can be inferred by selecting the *K* value that has the lowest PAC.

### Acquisition of two testing datasets

#### Gene mutation data for gastric cancer

We obtained somatic gene mutation data of 437 gastric cancer patients through the Xena Browser (https://xenabrowser.net/datapages/) for The Cancer Genome Atlas (TCGA), which is a landmark cancer genomics program that molecularly characterized over 11,000 cases of primary cancer samples. Based on the work of Dechao Bu et al. [22], we removed 35 samples missing survival information and 71 samples with high tumor mutation burden (TMB) and screened out 69 genes that were actionable. Finally, we have constructed a matrix with dimensions of 69 rows and 331 columns. Each row represents a gene, and each column represents a patient. The non-zero elements in this matrix account for approximately 1.63%, clearly indicating that it is a sparse matrix.

#### Gut microbial data for colon cancer

Gut microbiota abundance and composition affect the occurrence and progression of colorectal cancer, which can be used for subtyping of colorectal cancer patients. We obtained the gut microbial data of 195 colon cancer patients through the The Cancer Microbiome Atlas (TCMA, https://tcma.pratt.duke.edu/), which is a collection of curated, decontaminated microbial compositions for multiple types of cancers. Finally, we have constructed a matrix with dimensions of 221 rows and 195 columns, where each row represents a gene, and each column represents a patient. The non-zero elements in this matrix account for approximately 3.96%, fully demonstrating its sparsity.

### Assessing coefficients

#### Entropy

In cluster analysis, the concept of entropy is used to assess the stability and reliability of the clustering results [23–26]. When entropy is small, it means that most of the samples are clustered in one large cluster, while the others are grouped into a few small clusters [23]. In this case, the clustering results are less stable, as any point of perturbation or missing data may cause the samples that have been grouped into small clusters to be reallocated to the large clusters, resulting in large changes in the clustering results [27, 28]. In addition, the smaller number of samples in the small clusters results in the extracted features possibly lacking sufficient representation, reducing the reliability of the classification. Conversely, when the entropy value is large, it indicates that the number of samples included in each classification is relatively large, and therefore the classification results are more stable and more reliable. Therefore, entropy is widely used in cluster analysis to assess the quality and stability of clustering results [29].

We can calculate the entropy of the classification results for dataset $D$ in this way [26]:

8 $$Ent\left( D \right) = \mathop \sum \limits_{k} p_{k} log\left( {p_{k} } \right)$$

where $p_{k}$ is the probability of the sample being classified into the Kth cluster.

#### Calinski–Harabasz index

The Calinski–Harabasz index (CHI) is an internal evaluation metric for cluster analysis, designed to measure the tightness and separation of clustering results [30–33]. It is calculated based on the intra-class and inter-class variance of the clusters, allowing assessment of the quality and effectiveness of the clustering. In calculating the intra-class variance, the metric takes into account the sum of the squares of the distances from each sample point to the center of the class to which it belongs, i.e., the intra-class sum of squares, with smaller values indicating tighter data points within the class. When calculating the between-class variance, the metric takes into account the sum of the squares of the distances from the centroid of each cluster to the center of the entire data set, i.e., the between-class sum of squares, with larger values indicating greater distances between different clusters, i.e., better separation between clusters [34]. Therefore, the Calinski–Harabasz index is a metric for evaluating the quality of clustering based on the ratio of the intra-class sum of squares to the inter-class sum of squares, where a higher index value indicates better quality of clustering results.

We can calculate the Calinski–Harabasz index in this way [34]:

9 $$s = \frac{{tr\left( {B_{k} } \right)}}{{tr\left( {W_{k} } \right)}} \times \frac{{n_{E} - k}}{k - 1}$$

10 $$W_{k} = \mathop \sum \limits_{q = 1}^{k} \mathop \sum \limits_{{x \in G_{q} }} \left( {x - c_{q} } \right)\left( {x - c_{q} } \right)^{T}$$

11 $$B_{k} = \mathop \sum \limits_{q = 1}^{k} n_{q} \left( {c_{q} - c_{E} } \right)\left( {c_{q} - c_{E} } \right)^{T}$$

where $n_{E}$ is the number of training samples, *k* is the number of categories, $B_{k}$ is the between-category covariance matrix, $W_{k}$ is the within-category data covariance matrix, and $tr\left( \cdot \right)$ is the trace of the matrix.

### Implementation

PWSC is available as a open source software package for the R programming framework. It relied on the R packages cluster, clusterSim, pheatmap, ConsensusClusterPlus, fpc, clv, clvalid. To facilitate its application, we have developed a user-friendly web tool that implements the PWSC algorithm, which is constructed using the Python Flask framework. It takes Nginx as a reverse proxy server to handle a large number of concurrent requests. AJAX dynamic data, REACT frontend framework, and Ant-design component library are used to create user-friendly layout and visualizations. The PWSC web server is now hosted on an elastic cloud server from the Aliyun Cloud running an Centos Linux system (7.9.2009 with 16 CPU and 32 GB memory. It can be accessed at http://pwsc.aiyimed.com/from any platform by using modern Web browsers (recommended but not limited to the latest version of Safari, Chrome and Firefox).

## Result

### Framework of PWSC

**Fig. 1:**
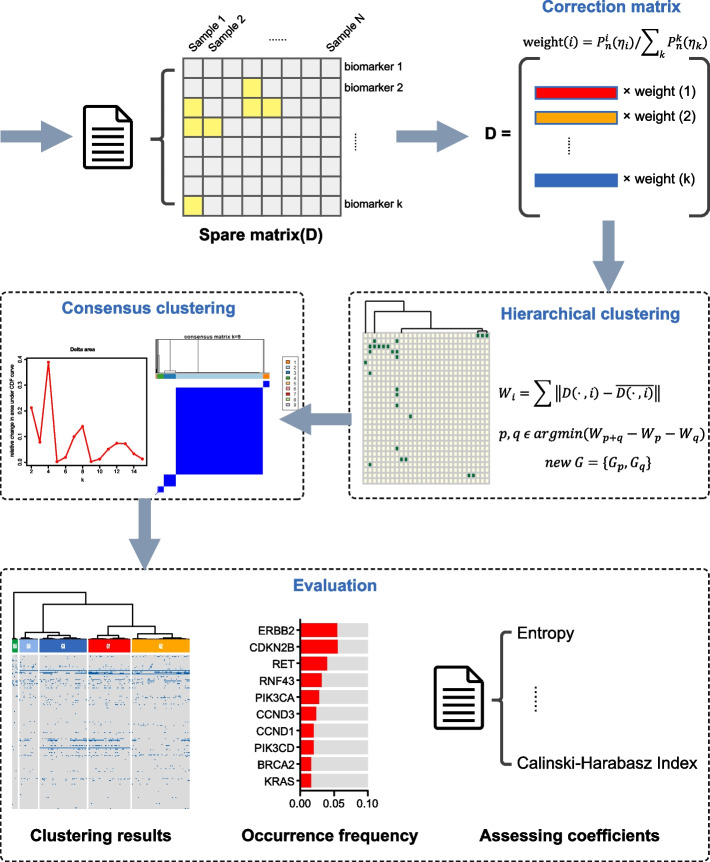
Frame work of PWSC

The Fig. 1 shows the procedure of PWSC. Data pre-processing is first performed to obtain a sparse matrix, which is used as input to the clustering algorithm. Then, a polynomial $P_{n}^{i} \left( {\eta_{i} } \right)$ is defined to calculate a correction matrix D, which is used to more accurately represent the degree of affinity between different samples. The correction matrix is clustered using the hierarchical clustering algorithm and the quality of the clustering results is assessed by means of CDF plots and consensus matrix heatmaps to select the best number of clusters. Finally, the clustering heatmap was redrawn and the occurrence of different genes in each cluster was counted to identify the most valuable genes in each cluster, while the clustering results were assessed using assessing coefficients such as Calinski–Harabasz index, entropy, etc.

### Subtyping application on tumor mutational data

#### Clustering results and assessing coefficients

**Fig. 2:**
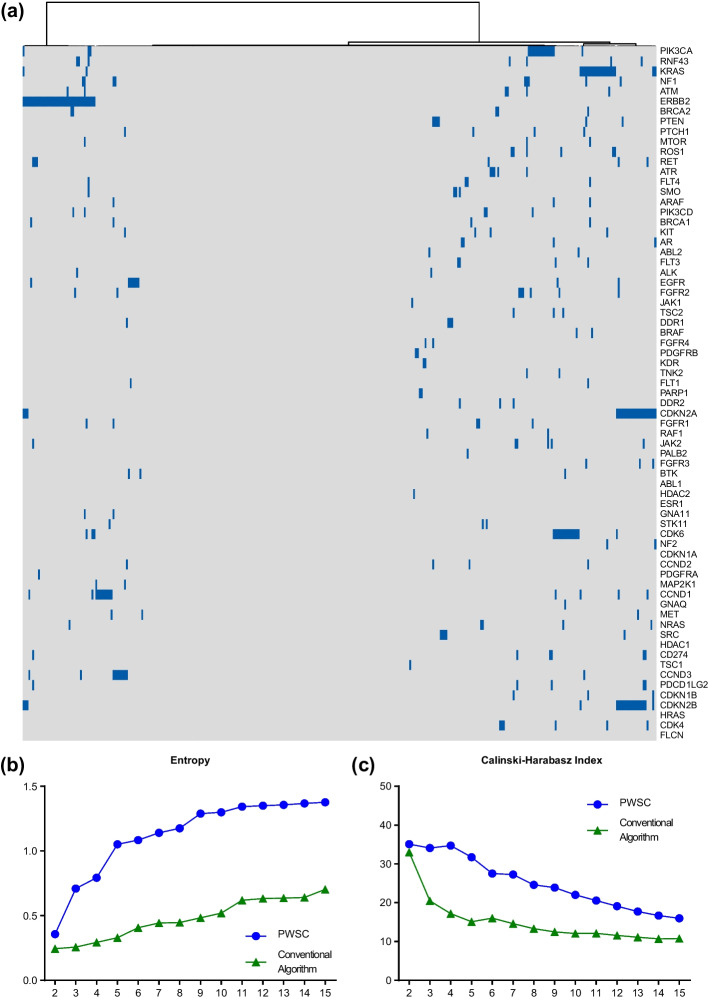
Clustering results and assessing coefficients. **a** The clustering heatmap of biomedical data. **b** The Entropy of PWSC and conventional algorithm when k is from 2 to 15. **c** The CHI of PWSC and conventional algorithm when k is from 2 to 15

We applied the PWSC algorithm to perform cancer subtyping on a cohort of 331 gastric cancer patients. The orginal input sparse matrix contains the specific gene mutations carried by each patient. We set the weight function $P_{n}^{i} \left( {\eta_{i} } \right) = \eta_{i}^{4} , \overline{\eta }_{i} = \frac{{\eta_{i}^{4} }}{{\mathop \sum \nolimits_{k = 1}^{m} \eta_{k}^{4} }}$ brought into Algorithm 1 for clustering calculation. The clustering results obtained are shown below. By looking at the clustering heatmap (Fig. 2a), we found that some of the genes would be concentrated in a certain region in the clustering heatmap, which indicates that these genes have an important role in determining the clustering results, and they are likely to be an important basis for dominating our clustering.

In addition, we observed the entropy values and the results showed that the PWSC algorithm also had a more significant increase in entropy values compared to the conventional algorithm of utilizing euclidean distance (Mann–Whitney U-test, W = 186, *p* value = 2.905e−05, Fig. 2b), which indicates that the clustering results of PWSC are more complex, stable and have better clustering results [35, 36].

Next, we calculated the Calinski–Harabasz index when the number of classifications k took on a range of values from 2 to 15. This is shown in Fig. 2c below. The analysis shows that the Calinski–Harabasz index values for the same number of classifications are significantly higher when using the PWSC algorithm for clustering compared to the conventional algorithm (Mann–Whitney U test, W = 192.5, *p* = 0.0004871), which indicates that the PWSC algorithm can perform better clustering analysis and improve the accuracy and reliability of clustering analysis [35, 36].

#### Best clustering results

**Fig. 3:**
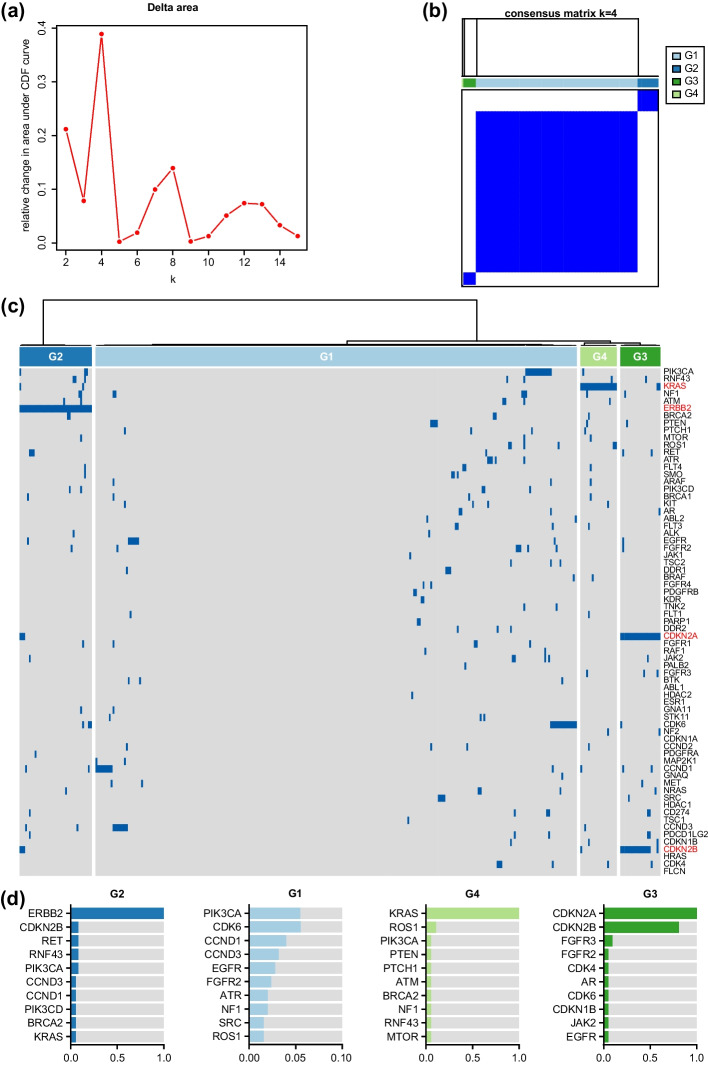
Optimal clustering and occurrence of genes for the gastric cancer cohort. **a** The consensus clustering CDF curve when k is from 2 to 15. **b** The consensus matrix when k is 4. **c** The clustering heatmap of gene mutations for four groups. **d** The most valuable genes with high occurrence in each group

We combined the consensus clustering approach with the above methods to calculate the consensus CDF curve (Fig. 3a) and the consensus matrix (Fig. 3b). Upon analyzing the curve, we found that it peaked at k = 4, 8, and 13. Considering the cost of clustering, a larger number of classifications can lead to a less stable clustering result [37]. Therefore, we selected the 4-cluster classification as the optimal number of classifications based on the Calinski–Harabasz index, which reached its maximum value at k = 4 and was significantly higher than at k = 8 and k = 13.

Furthermore, the consensus matrix heatmap was very clear at k = 4, indicating that the probability of each sample being misclassified during the consensus clustering test was low, demonstrating the high stability of the clustering at this point [21]. Thus, selecting k = 4 as the optimal number of clusters is appropriate. A remarkable 100% improvement in the quality of the best classification were observed compared to conventional algorithms (Fig. 2c).

We divided the clustering results based on the distance between the individual clusters, dividing the samples into four groups and presenting the results as a clustering heatmap shown in Fig. 3c. We found that this result further validated our findings in the previous section: genes with a concentrated distribution were present in some of the groups and these genes played an important role in determining the grouping.

Finally, to investigate the practical implications of this classification, we counted the number of occurrences of genes in each group and identified those genes that were significantly different from other genes as most valuable genes. The analysis of Fig. 3d shows that ERBB2, KRAS, CDKN2B and CDKN2A are the most valuable genes in Group1, Group3 and Group4, respectively, which are all the hot oncogenes in gastric cancer. They have a significant difference in occurrence compared to other genes; while Group2 shows a mixed distribution of multiple genes, with multiple genes playing a role in the patient's disease [38–40]. In contrast, Group2 showed a mixed distribution of genes, with multiple genes playing a role in the disease.

Through the analysis of the most valuable genes, we can see that, on the one hand, PWSC can extract the genes that are dominant in the clustering, and through the extraction of these genes, we can have a deeper understanding of the disease, and also achieve more precise treatment according to the different gene expression of the patients [41–45]. The PWSC can help identify the most valuable biomarkers. These biomarkers are not only essential factors for classifying different groups, but also cover a wide scope of instances.

### Subtyping application on tumor microbial data

The gut microbiome is a key player in the immunomodulatory and protumorigenic microenvironment during colorectal cancer (CRC), as different gut-derived microbes can induce tumor growth. Thus, it has been used for subtyping of colorectal cancer patients. We applied the PWSC algorithm on the original input sparse matrix, which contain the 221 microbial abundance for 195 patients, with 3.96% of non-zero elements.

**Fig. 4:**
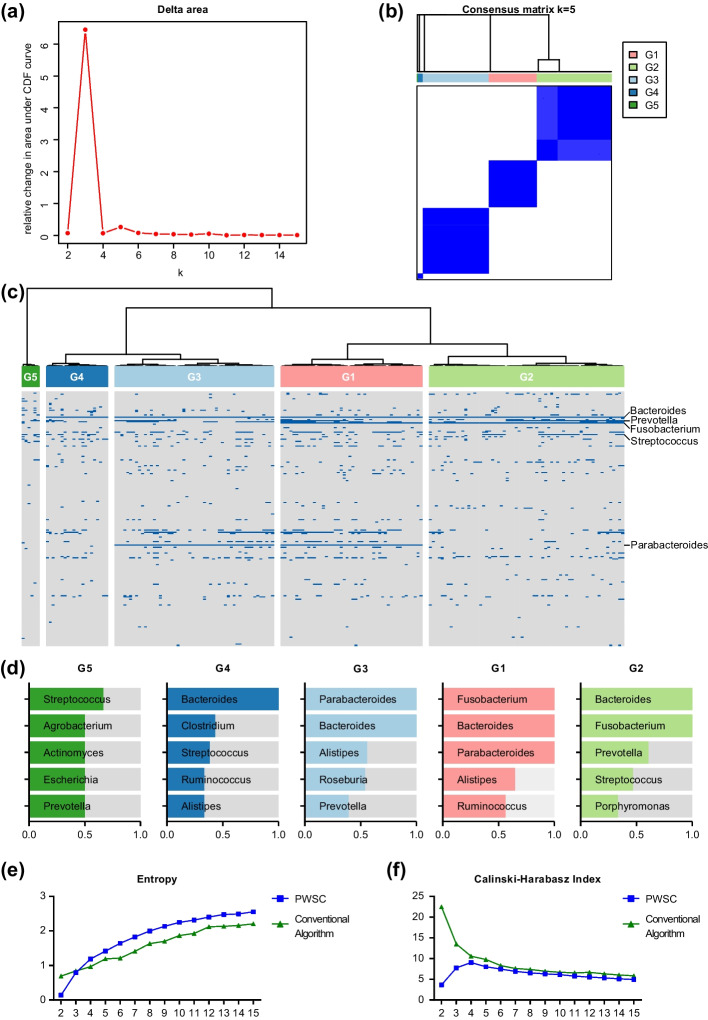
Subtyping of colorectal cancer cohort using tumor microbial data. **a** The consensus clustering CDF curve when k is from 2 to 15. **b** The consensus matrix when k is 4. **c** The clustering heatmap of microbial data for five groups. **d** The most valuable biomarkers with high occurrence in each group. **e** The Entropy of PWSC and conventional algorithm when k is from 2 to 15. **f** The CHI of PWSC and conventional algorithm when k is from 2 to 15

Considering the relative change in area under CDF curve (Fig. 4a) and visiualization of consensus matrix (Fig. 4b) for each K, we selected the 5-cluster classification as the optimal number of classifications. Under this classification, G1 contains 48, G2 contains 66, G3 contains 54, G4 contains 21, and G5 contains 6 samples (Fig. 4c). We have tallied the occurrence of microorganisms in each group and found that different groups are dominated by quite different bacteria. Bacteroides is a genus of bacteria that naturally exists in the microbiota of the human gut. They are highly present in G1–G4, but not in G5. Fusobacteriums are highly present in G1, G2, but not in G3, G4, G5. Parabacteroides are highly present in G1, G3, while not in G2, G4, G5 (Fig. 4d).

Next, we calculated the entropy values and Calinski–Harabasz index when the number of classifications k took on a range of values from 2 to 15. A significantly increased Entropy (*p* = 0.0336) (Fig. 4e), as well as comparable CHI (Fig. 4f), were observed compared with the conventional algorithm. The above attempts in cancer subtyping demonstrate that PWSC is highly applicable to different types of biomedical data.

### Online web service

**Fig. 5:**
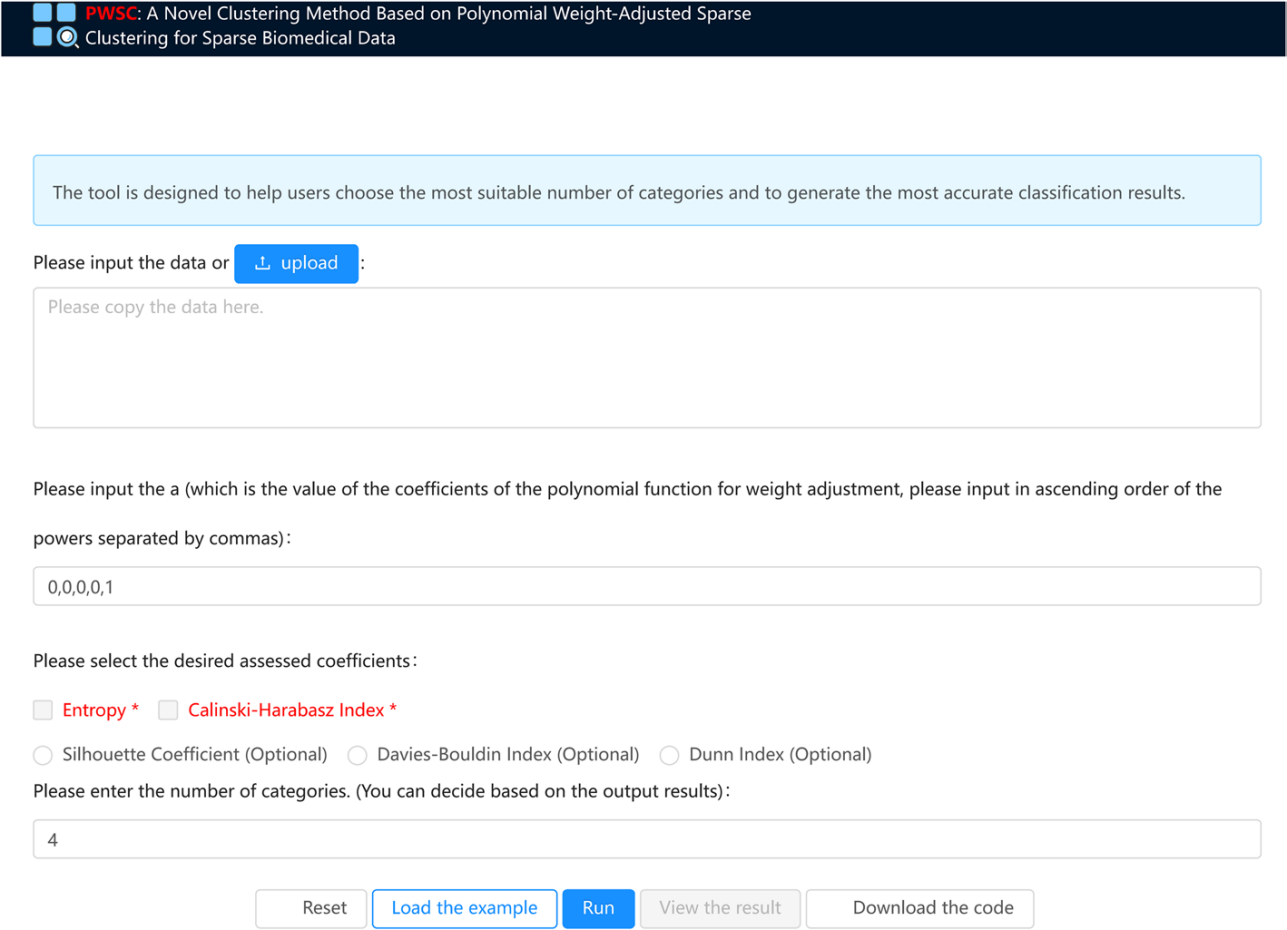
Online web service interface presentation

To make it easier for users to utilize our accomplishments, we have created an online web service (Fig. 5). Users are required to input the data that needs to be clustered and choose the desired assessing coefficients. We will then generate the corresponding consensus clustering results and assessing coefficients. Based on these results, users should enter the number of classifications that fulfill their specific requirements. Lastly, we will produce the clustering result heatmap and the corresponding most valuable biomarkers for each group.

The PWSC website utilizes the NGINX service as its power source. The methods have been refactored into RESTful APIs, and AJAX is used to dynamically refresh data on the web page. To enhance accessibility, we have adopted the REACT frontend framework, along with the Ant-design component library to create user-friendly layouts and display data tables. In addition, Echarts is utilized for interactive chart display.

You can upload the biomedical data that needs to be clustered with the assistance of PWSC. Then, select the desired test coefficients and input the number of categories to be tested. Finally, click on 'Run' to obtain the clustering results. Alternatively, you can click on 'Load the example' to view an example. The data in the example is the experimental data of this article, and all experimental results can be downloaded after running the example. Additionally, for your convenience in further research and study, you can click on 'Load the code' to access the program's code. This website can be accessed through http://pwsc.aiyimed.com/.

## Discussion

When clustering sparse matrices, we observed that the sparsity of the data matrix leads to a less compact distribution of sample points in the high-dimensional space characterized by the data. It becomes challenging to find a consensus on an effective partition criterion to assign some samples to a specific category. In hierarchical clustering, the principle of clustering is to assign samples with close “relatedness” to the same category and those with distant “relatedness” to different categories, thus studying the relationship between samples. However, when samples exhibit sparse distribution in space, it means that the "relatedness" between samples is distant, making it difficult to find a closure to partition samples with certain features from the original samples [46]. This results in conventional clustering methods being unable to classify samples in biomedical research when the data matrix is sparse, making it impossible to conduct targeted studies on samples.

The article is based on this point and proposes the use of PWSC to solve this problem. The advantage of PWSC lies in its ability to better separate data by modifying the data matrix through the establishment of polynomial weights. Compared with conventional algorithms and methods that directly use gene occurrence frequency as weights, PWSC can make samples of the same class more compact and those of different classes more dispersed, thus more accurately reflecting the “relatedness” between data. This data modification method can improve the accuracy and stability of clustering results.

On the one hand, PWSC uses polynomial functions for weight modification, avoiding the potential problem of numerical overflow when using the Soft-max function as weights. By manually adjusting the polynomial function, the function value and adjusted weight value can always be kept within a suitable range, avoiding numerical overflow and underflow and ensuring the stability and reliability of the algorithm [47, 48]. On the other hand, compared with the exponential function (Soft-max function), the polynomial function has a slower growth rate. This means that when using the polynomial function to process weights, the differences between data points will not be overly magnified, effectively avoiding the problem of data points being overly stretched and resulting in poor clustering results. This weight modification method can more reasonably and controllably handle differences between data points, thereby improving the effectiveness and stability of clustering.

At the same time, we used the method of consensus clustering to determine the optimal number of clusters. On the one hand, this can help us avoid the influence of subjectivity and subjective bias on the selection of the number of clusters, thus determining the number of clusters more objectively and accurately. On the other hand, it can better reflect the stability and consistency of clustering results under different numbers of clusters. By considering the results of consensus clustering, interference caused by fluctuations in clustering results due to noise or randomness can be avoided in the selection of the number of clusters.

From the classification results, we can see that the classification results of the PWSC algorithm can help researchers identify prominent genes in different groups, known as “most valuable biomarkers”. By statistically analyzing the occurrence of genes, significant differences between genes in different groups can be determined, thus studying the role of different genes in the process of causing disease in humans and helping us to better understand the mechanism of disease occurrence [49]. This can help doctors make more accurate treatment decisions based on the patient's gene expression. Additionally, the PWSC algorithm can assist doctors in distinguishing between disease caused by gene mixing and disease caused by a single gene. By comparing with other groups, PWSC can help doctors better understand the role of different genes in the patient's disease process, thus more accurately classifying and diagnosing patients.

In summary, the PWSC algorithm has the advantages of improving the accuracy, stability, and reliability of clustering analysis through the polynomial weighting correction method. Additionally, the PWSC algorithm can help researchers gain a deeper understanding of the mechanism of disease occurrence and assist doctors in making diagnoses.

## Conclusions

PWSC algorithm provides an effective solution for handling sparse biomedical data. By utilizing the PWSC algorithm, researchers can accurately classify genes and identify "star genes" that play a significant role in disease mechanisms. This helps us gain a deeper understanding of the underlying causes of disease and provides valuable insights for medical professionals to make more precise treatment decisions based on a patient's gene expression patterns. With the ability to handle sparse biomedical data and identify important genes, the PWSC algorithm holds great potential in advancing our understanding of disease and improving patient outcomes.

## Data Availability

Genomic data of gastric cancer patients are downloaded from the Xena Browser with identifier of TCGA-STAD (https://xenabrowser.net/datapages/).
